# Using School-Based Teleconsultation Services to Make Community Health Services Accessible in Semirural Settings of Pakistan: Sequential Explanatory Mixed Methods Study

**DOI:** 10.2196/48664

**Published:** 2024-11-12

**Authors:** Saleema Gulzar, Shirin Rahim, Khadija Dossa, Sana Saeed, Insiyah Agha, Shariq Khoja, Rozina Karmaliani

**Affiliations:** 1 School of Nursing and Midwifery Aga Khan University Karachi Pakistan; 2 Aga Khan University Hospital Karachi Pakistan; 3 Department of Pediatrics and Education Development Aga Khan University Hospital Karachi Pakistan; 4 Department of Pediatrics Aga Khan University Hospital Karachi Pakistan; 5 Tech4Life Enterprises Ontario, ON Canada

**Keywords:** teleconsultation, digital health, school health, child health, information technology, eConsultation, telehealth

## Abstract

**Background:**

In Pakistan’s remote areas, quality health care and experienced professionals are scarce. Telehealth can bridge this gap by offering innovative services like teleconsultations. Schools can serve as effective platforms for introducing these services, significantly improving health service access in semirural communities.

**Objective:**

This study aims to explore the feasibility of introducing school-based teleconsultation services (TCS) to strengthen community health in a semirural area of Karachi, Pakistan.

**Methods:**

This study used a mixed methods design. A total of 393 students were enrolled for the quantitative component, while 35 parents, teachers, and community stakeholders participated in the qualitative arm (focused group discussion). Proportional computation for the quantitative data was done using SPSS (version 24; IBM Corp), while qualitative data underwent thematic analysis.

**Results:**

A total of 1046 successful teleconsultations were provided for 393 students over 28 months. The demographic data showed that the mean age of the students availing TCS was 9.24 (SD 3.25) years, with the majority being males (59.3%, 233/393). Only 1.24% (13/1046) of cases required referrals. The qualitative analysis yielded three themes: (1) transformation of the health care experience, (2) escalating demands for teleconsultation, and (3) the psychological aspect of care.

**Conclusions:**

This study demonstrated the efficacy of integrating TCS in a semiurban school in Karachi to address health care accessibility gaps. Implementing TCS through the school platform improved the overall health status of school children while reducing school absences and financial burdens on families. The study highlighted TCS’s cost-effectiveness, time efficiency, and quality, with community support for 24/7 availability, expansion to adults, and a reimbursement model. School health nurse-led TCS offers a scalable solution to health care challenges, enhancing health outcomes for school-going children in Pakistan and globally, particularly in low- and middle-income countries, where accessibility is a major issue.

## Introduction

Health care inaccessibility remains a persistent concern in transitioning regions, even in the 21st century [1,2]. Pakistan is one of the countries in Asia where remote areas lack access to experienced health professionals who could provide quality health care services and information [3]. The lack of quality health care compels people from remote areas to seek help from unqualified practitioners [4], which can lead to further complications. First, there is an unavailability of safe and quality health care services in remote urban and rural areas as physicians and nursing cadres are unwilling to live in these areas due to poor health facilities and limited lucrative opportunities. Second, since patients live at considerable distances from major cities, they spend excessive amounts of money to reach hospitals and clinics for both serious and nonserious ailments [1-4]. Furthermore, in many cases, a male family member skips work to take someone for medical consultation, hence losing that day’s earnings. Finally, the lack of an organized public health care system in Pakistan puts the entire burden on the few available public hospitals [1-5]. The global community has committed to certain benchmarks, formerly as Millennium Development Goals (MDGs) and recently as Sustainable Development Goals (SDGs), for eradicating poverty, protecting the environment, empowering communities, and ensuring healthy lives and well-being for all ages. To meet the SDG goals, it is critical to provide health care services and information to people in their own communities. Digital health can play a major role in supporting conventional health services to fulfill this demand in distant semiurban and rural areas of Pakistan.

Digital health is a rapidly growing field, and the adoption of telehealth services is becoming increasingly common [1,6]. While Digital health is widely used in high-income countries, it is not readily available to the masses in Pakistan. It is a convenient and helpful service that can help reach more secluded areas of Pakistan. Its wider adoption would benefit society by allowing access to health care regardless of distance and economic status. Digital health solves logistical barriers, supports weak health systems, and helps establish worldwide networks of health care professionals [6].

Digital health is defined as “the cost-effective and secure use of Information and Communication Technology (ICT) to support health and health-related fields, including healthcare services, health surveillance, health literature, health knowledge, and research” [7]. It has emerged as an important source for enhancing access to health and information and improving response time to matters impacting personal or community health [8]. Digital health enhances the delivery of health care services where treatment cost and distance are critical factors [8]. Considering digital health solutions, telehealth through teleconsultation services (TCS) is a viable option for making health care accessible [9]. This study was undertaken to bring health care services to the community’s doorstep and address the health needs of school-aged children through TCS in a poor semiurban settlement in Karachi, Pakistan. This project was tied with the school health program at a local government school using a nurse-led model to facilitate teleconsultation of school children with a physician available virtually.

Developing a teleconsultation service as a school health program was an innovative idea to make health care accessible to people living in far-flung semiurban communities where health care facilities are not easily available [10]. The school health program became the first step in exploring the feasibility of bringing health care to such communities through TCS [10]. The uniqueness of this project was that the selected school, which was initially nonfunctional, was adapted to convert it into a model educational institution where education and school health go hand in hand. The teleconsultation services added value to this innovation. From the beginning, the detailed educational, health, and IT needs of the potential students attending this school were assessed by a team of experts. The findings of the needs assessment helped the research team develop a plan to build a school health room with IT capabilities to initiate telehealth services, addressing the health needs of school-going children—one of the vulnerable populations. This initiative served as a prototype school health service catering to the target population and other areas where access to health is difficult or nearly impossible. Once the feasibility of TCS at this school is proven, the project could be scaled up for a wider community and more health conditions.

Using technology in school health services is essential for improving efficiency, communication, and student well-being [10,11]. It streamlines administrative tasks, improves communication among health professionals, students, and their parents, and ensures accurate record-keeping. Real-time health monitoring and telehealth services enhance timely interventions, especially in remote areas. Technology enables data analysis for health trends, supports health education, and fosters active parental involvement [11]. This study will be a stepping stone for scaling up the same for the community at large and for other parts of Pakistan. Therefore, the aim of the study is to explore the feasibility of introducing school-based TCS to strengthen community health in a semiurban area of Karachi, Pakistan. We hypothesized that the accessibility of students and their parents to health services and information would increase through the implementation of school-based TCS. In addition, the qualitative arm of the study would provide insight into the usefulness and feasibility of TCS implemented through school health settings.

## Methods

### Study Design

A sequential explanatory mixed methods design was used to assess the feasibility of TCS at the local government school in the semi-urban setting of Karachi, Pakistan. The rationale for selecting the mixed method approach was to provide a holistic perspective of the TCS initiative undertaken.

### Study Setting

The study was conducted at a government school in a semi-urban community in Karachi, serving 34 villages within a 10-km range. The total number of households in these villages was 2052, with approximately 10,750 inhabitants. The commonly spoken languages of the community were Balochi and Sindhi. Male members of the community contributed to household income by working as laborers and farmers. Some were also involved in military and protective services. The majority of married females were stay-at-home spouses, with a few working in the health and education sectors. The catchment population opted for private sector services due to the lack of public sector facilities in the area, which added financial and time burdens for the local community.

A nonfunctional public school in the aforementioned semiurban setting of Karachi, Pakistan, was adopted by a nongovernmental organization (NGO) in partnership with the government, aiming to turn it into a model school with all the necessary components of an ideal educational institution. The school health service, being a mandatory element of any ideal school, was brought to this model school through the current project to provide health services to children attending the school.

The reason for selecting this particular school was 2-fold: first, it was situated in a semiurban setting with almost no access to health care; second, the school was in a growing phase with a lot of potential for adapting new initiatives. The partnership between the government and an NGO helped the project team introduce and execute a nurse-led school health project with the innovation of telehealth services. As the school grew, it offered education from ECD (Early Childhood Development) to grade 8 by a team of 7-8 teachers, including 1 male and 6 female teachers, all from the same community. The student body of the selected school predominantly consisted of males, with a male-to-female ratio of 60:40.

The population served by this project was school students from a semi-urban settlement in Karachi. The service was open to all students attending the school and falling sick during school hours.

### Tele Consultation Service

For this study, a “Hub and Spoke” model [12] was used in one of the local government schools with 393 enrolled students. The school served as a spoke, connected to the Pediatric Department of a private University Hospital in Karachi, which acted as the hub for providing TCS for the children studying at the school.

For this study, the use of TCS was limited to students attending the selected school during official hours (0900-1200) for health issues related to children aged 5-14 years. All students feeling unwell were allowed to avail themselves of the TCS. The average turnout was 3-4 students per day. The services provided during TCS included the following:

1. Recording the demographic data of the students (name, age, gender, grade, presenting complaint, height, and weight).
2. Conducting a complete initial assessment of the student, including history, vital signs, and physical assessment related to the presenting complaint, by the school health nurse using digital equipment (stethoscope and dermascope).

This information was reviewed by the pediatrician, who provided appropriate advice for diagnosis and treatment using TCS.

The TCS at the school operated in 2 ways:

1. Live synchronous consultations: these occurred twice a week when the pediatrician was available online for 3 hours. During this time, the pediatrician attended to students for their initial visits and all follow-up visits. The entire assessment and consultation were recorded in the system by the nurse.
2. Store-and-forward consultations: these took place on the days when the pediatrician was not available live. In this mode, the nurse uploaded the student’s assessment into the system and sent an alert to the pediatrician. The pediatrician would review the initial assessment of the system. If additional information or further assessment was needed, the pediatrician would consult the nurse. The nurse would then upload images and data from digital equipment such as a stethoscope, ophthalmoscope, and dermascope. Once the complete assessment was reviewed, the pediatrician proposed the treatment, which the school health nurse then uploaded to the system.

After each consultation (live or store-and-forward), the school health nurse conveyed all the medical advice suggested by the pediatrician to the parents of the children seeking TCS and maintained regular follow-ups on all cases as per the pediatrician’s comments and advice.

The privacy of all students availing TCS was ensured by uploading all data onto a password-secured online system, accessible only by the school health nurse, the pediatrician, and the principal investigator. Snapshots or assessments were taken using digital equipment connected to the online system and were directly uploaded.

The duration of each consultation varied depending on the presenting complaint and the nature of the visit (initial or follow-up). An average initial visit took approximately 30 minutes, while an average follow-up visit took about 15 minutes. The pediatrician received a minimal token payment of PKR 8000 (approximately US $30) per month for participating in the TCS program.

### Teams and Infrastructure

An infrastructure for TCS was established at the target school by developing a health assessment room, with a school health nurse available physically and a pediatrician joining remotely. Various partners actively participated in executing the project, including members from a private School of Nursing and Midwifery, the administration and IT staff of the local school, telehealth specialists from a private digital health firm in Karachi along with their support staff, a school health nurse who was a trained Lady Health Visitor (LHV), and a trained pediatrician.

### Recruitment of Study Participants

The study participants for the quantitative arm were recruited through total or universal sampling, including students of all ages and grades, from ECD to grade 8, attending the school (N=393).

For the qualitative arm, 35 participants were recruited through purposive sampling. These included community stakeholders, teachers, and parents of students who used the TCS at the school during the study period and were willing to participate.

A formal launch of the TCS was executed in 2017 at the target school through a ceremony in which all relevant community and school stakeholders, parents, teachers, and related government officials were invited. The entire process of live teleconsultations was demonstrated at the launch, with the school health nurse presenting a case to the pediatrician attending the consultation online. Through this event, the community and parents were mobilized, and services were formally initiated for the school.

Parents who attended the event were informed about the research project and asked for their consent. The day after the event, the school health nurse visited each class to explain the project, the role of the teleconsultation service, the online physician, and the nurse in managing minor ailments of the students. A meeting was also held with the school teachers to explain the project and instruct them to direct students who fell ill during school hours to the health room for immediate teleconsultation services.

### Data Collection and Analysis

Data was collected during April 2017 and January 2020. This excludes summer and winter vacations, weekends, and public holidays. Hence, the total time duration in which TCS was provided was 17 months.

#### Quantitative Data

Quantitative data included demographic information such as the age and sex of the participants, as well as monthly data on the number of successful teleconsultations, referrals, follow-ups, complaints for which TCS was sought, and types of treatment received. This data was retrieved from the monthly logbooks maintained by the school health nurse and analyzed using descriptive statistics with SPSS (version 24; IBM Corp).

#### Qualitative Data

Qualitative data were collected through focus group discussions (FGDs) using a semistructured interview guide. In total, 3 separate FGDs were conducted at the school with 12 parents, 6 teachers, and 4 community stakeholders. The FGDs were audio-recorded with the participants’ permission and later transcribed for analysis. Each FGD lasted approximately 45-60 minutes. The community stakeholders included community leaders who made themselves available for the discussions.

The broad topics considered during the discussions included the need for TCS services, the benefits of TCS services, challenges, and ways to improve TCS at the school. The recordings were transcribed immediately after the discussions to ensure trustworthiness. The data underwent manual content analysis, with each meaningful sentence taken as the unit of analysis. Through this process, codes were identified, and similar codes were clumped together to generate subthemes, from which unique themes were extracted [13]. Data collection continued until saturation was achieved.

The study used the following steps for analyzing the qualitative data.

Data analysis commenced by transcribing audio-taped interviews verbatim and translating them into English. These transcripts and translations were assigned unique code numbers and formatted uniformly in a Microsoft Word document with 5 columns: transcription, translation, code, categories, and themes. They were meticulously organized in both ring binders and electronic folders.

Thorough immersion in the data was achieved through multiple readings of the transcripts to attain a profound understanding and description of the phenomenon. During this phase, each paragraph of every transcript was systematically coded. Similar ideas within each transcript were consolidated under corresponding codes. These codes, with similar meanings, were subsequently amalgamated to form categories.

Common themes were derived by scrutinizing all categories for connections and similarities. To substantiate each theme, pertinent quotes from participants were extracted from the transcripts and linked to each theme, accompanied by the participant’s code.

These methodical steps ensured a comprehensive and systematic analysis of the qualitative data collected.

The identified themes were interpreted as the study’s findings and underwent verification for integrity and reliability by research team members. Furthermore, these themes were compared with existing literature to discern similarities and differences in the ideas uncovered by the study.

### Ethical Considerations

The study received approval from the Ethical Review Committee of Aga Khan University (AKU-ERC 4410-SON-ERC-16) in November 2016 and was conducted from January 2017 through December 2020.

Written informed consent was obtained from all parents, and verbal consent was obtained from all children who participated in teleconsultations. Participants were informed about the study’s purpose, process, and benefits and were given the option to participate voluntarily and withdraw at any stage.

Privacy and confidentiality were strictly maintained by assigning unique code numbers to each participant using TCS. Participant information was password protected, and access was restricted to the core research team, which included student nurses and physicians.

No monetary compensation was provided to the students, as teleconsultations were conducted within the same schools where the students were enrolled.

## Results

### Quantitative

Over a span of 17 months, a total of 1046 teleconsultations were conducted, serving 393 students. Among these consultations, 37.57% (393/1046) were initial visits, and 62.42% (653/1046) were follow-ups. The majority of disease-related complaints were effectively managed through TCS, with only 1.24% (13/ 1064) necessitating referrals to specialized services (Table 1).

The demographic data revealed that students using TCS had a mean age of 9.24 (SD 3.25) years, with a majority being male (59.3%, 233/393 students).

**Table 1:** Number and proportions of initial visits, follow-up, and referrals out of the total 1046 TCS offered to 393 students.

	Students, n (%)
Initial visit	393 (37.5)
Follow-up	653 (62.42)
Referral	13 (1.24)

The health issues for which TCS was provided were categorized into the following subgroups: dermatology; eye, ear, nose, and throat (ENT); gastrointestinal; hematology; musculoskeletal; oral health; and upper respiratory tract. Among these subgroups, the majority of cases were related to the upper respiratory tract (29.8%, 117/393 cases), followed by dermatology (20.6%, 81/393 cases), and musculoskeletal issues (19.1%, 75/393 cases; Table 2).

**Table 2:** Count and proportion of reported health issues during initial teleconsultation (N=393).

Health issues treated	Each health issue reported in total consultations (N=393), n (%)
Upper respiratory	117 (29.8)
Dermatology	81 (20.6)
Musculoskeletal	75 (19.1)
Ear, nose, and throat	55 (14)
Gastrointestinal	26 (6.6)
Oral Health	20 (5.1)
Eye	16 (4.1)
Hematology	3 (0.8)

**Figure 1:**
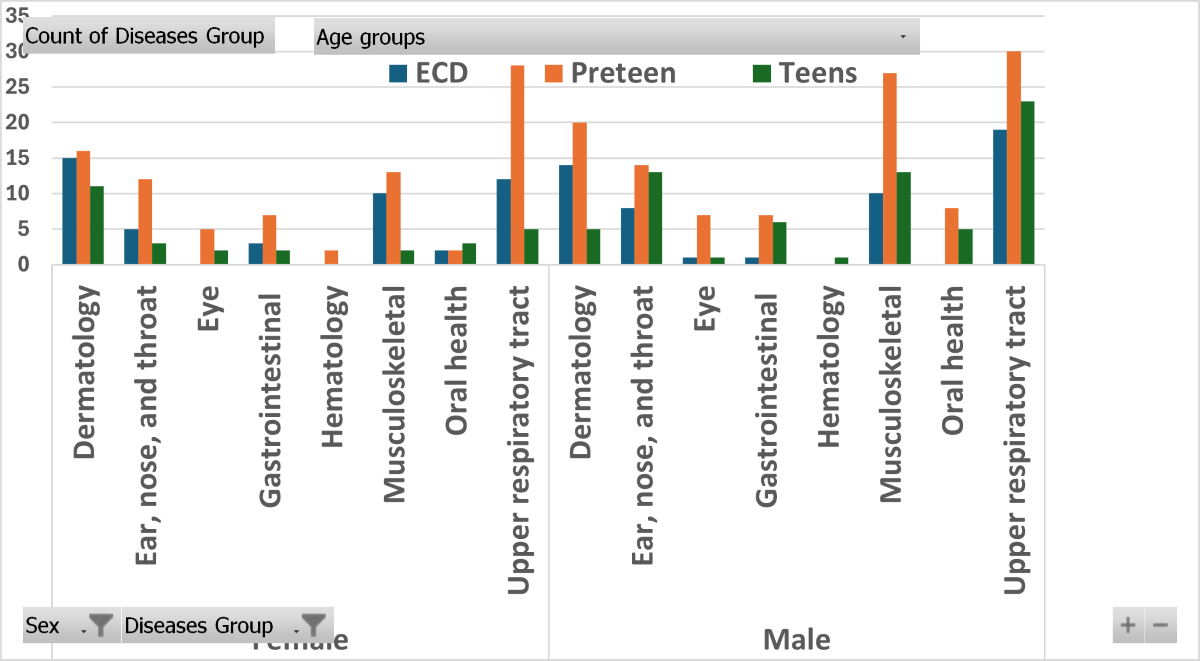
Gender and agewise distribution of reported health issues.

There was a distinct pattern of disease distribution according to the age and sex groups of the participants. The most frequently reported health issues among children of all ages and genders were related to the upper respiratory tract and musculoskeletal system, followed by dermatology and ENT issues. The distribution of diseases across age and sex groups is illustrated in Figure 1.

In addition, the treatment provided during teleconsultations revealed that the majority of students received topical medications (32%, 335/1046 cases), followed by oral antibiotics (20%, 209/1046 cases), and other medications (48%, 502/1046 cases) such as antipyretics and antihistamines. Essential medications were readily available either with the school health nurse or within the school vicinity.

### Qualitative

A total of three themes emerged from the analysis of focused group discussions: (1) the transformation of the health care experience, (2) escalating demands for teleconsultation, and (3) the psychological aspects of care.

#### Transformation of Health Care Experience

Parents or caregivers of students, as well as teachers, exhibited a fundamental understanding of TCS. The community began to view TCS as a convenient, cost-effective, and reliable health care service accessible right at their doorstep. TCS played a pivotal role in transforming health care experiences. Primarily, TCS provided the semi-urban community with access to essential health care services.

One parent stated the following:

We live in a village where healthcare facilities are not readily available, and it used to take hours or even a day to reach a doctor. Now, having online doctors available here is so convenient for us.

Another parent mentioned the following:

In our community, there are no doctors nearby. When our children get sick, the mother has to wait for the father to come from work to take them to a healthcare service that is located far away.

TCS also streamlined health care to be cost effective and time efficient. A caregiver expressed satisfaction with TCS, highlighting its affordability in terms of both money and time. They emphasized that these services enabled timely consultations with doctors, ensuring prompt management of their children’s health. Reflecting on the challenges the community faced before TCS, one parent shared the following:

Public transportation operates on a fixed schedule, and if someone falls ill afterwards, arranging private transport becomes very difficult or unaffordable due to high rental charges.

Another participant added the following:

“Previously, it took us hours to transport our sick children to healthcare services, but teleconsultation has made it much more feasible for us. A remote consultation used to cost us a minimum of two thousand rupees (approximately US$7).”

Participants expressed satisfaction with TCS for alleviating their challenges of seeking expensive and distant health care consultations for their sick children. One participant shared their contentment, stating the following:

This saves us time and money. When we travel for doctor visits, it wastes both time and money. This service also prevents our children from missing school; it's much better and convenient.

Furthermore, community members began to trust TCS for its quality, accessibility, and affordability. A parent highlighted their satisfaction, saying the following:

We are pleased that our child can receive medical treatment online at school. Now, they can stay healthy and continue their education under one roof. We are very satisfied with this service. Once, my child had a high fever; the nurse examined him, and the doctor prescribed medicine. By the grace of God, he recovered in three days.

#### Escalating Demands for Teleconsultation

The feasibility of TCS was widely acknowledged by members of the community. However, there were suggestions for enhancing these services, as caregivers and community leaders perceived the source as highly trustworthy and reliable. Participants expressed a desire for 24/7 TCS availability in their community to alleviate nighttime difficulties when children fall ill. One community leader voiced support for the service, stating the following:

It is a great initiative, however, there is a need for a 24/7 consultation service with an extended pharmacy service available also. The community leaders will arrange the financial resource and will pay for someone who is not able to afford the medicines.

Furthermore, participants expressed a desire for the expansion of TCS to include adults and other members of the community beyond just school-going children. They suggested broadening the scope to cover additional medical conditions such as delivery, as well as maternal and child health care, aiming to benefit the entire population.

One participant emphasized the following:

The facility should grow to encompass resources like injections, drips, nebulizers, oxygen, and basic lab tests. It should offer treatment for a wide range of conditions. There should be doctors available on-site. While the current consultation rooms are adequate, there should also be facilities for delivery, CT scans, and X-rays.

#### The Psychological Aspect of Care

During the analysis, several intriguing insights surfaced regarding the community’s perception of health care. The semiurban population primarily consisted of illiterate individuals who were unable to read, write, or communicate in any language other than the local Balochi or Sindhi. These individuals believed it was crucial to have a physician physically present to conduct a comprehensive assessment of sick patients and prescribe treatment accordingly. One community leader expressed dissatisfaction, stating the following:

Physical presence of the doctor will give us better satisfaction. We all are ill-literate here, some of us may understand about the online consultation but most of the community individuals will take this for granted.

Another participant shared that the following:

We cannot openly speak to the doctor online as we do face to face, which allows us to discuss health problems openly with experts.

One aspect that emerged from the FGD was people’s mindset and perception regarding the cost associated with health care and the preference for a physical examination by a doctor rather than teleconsultations. Study participants discussed the possibility of implementing a fee-for-service model for consultation services. They expressed that offering consultations free of cost might lead to them being undervalued and taken for granted. Given the challenges related to transportation and time loss in accessing health care, participants emphasized the importance of recognizing and appreciating TCS, which provides accessible and safe health care.

Caregivers suggested charging a nominal fee of one to two hundred rupees for these services, while some still preferred free consultations. One nurse remarked the following:

Nobody values free services. When they spend money, they care. Free-of-cost facilities are cared for by none. I think a small fee must be charged so that parents take some responsibility for the treatment of their children.

The same sentiment was echoed by another parent, who emphasized that the following:

Previously, when the child felt low, we had to take off from work and the child had to miss school to take the child to the doctor. But now, the facility is available here. So, parents send the child to the school nurse, or when we meet the nurse during the break, we tell her to call the child from class and see the child. For these many facilities here, the fees are moderate, and we have to give at least half of the fees.

## Discussion

### Principal Findings

Through this study, a total of 1046 teleconsultations were provided to 393 students over a span of 17 months. Among all the initial consultations, 62% (648 consultations) involved follow-up visits, which were continued till students availing TCS attained complete recovery.

Most of the cases involved respiratory infections, followed by skin problems, minor injuries, and ENT issues. During consultations, the process included assessing the child’s anthropometric measures, gathering details of the presenting complaint, and prescribing effective treatment regimens along with health education to the parents. The findings of health education have been published elsewhere [14].

Qualitative analysis revealed that participants had developed a basic understanding of TCS, finding it cost-effective and time-saving. They had begun to trust TCS for its quality, accessibility, and affordability. Suggestions for improving TCS emerged from the data, including providing 24/7 availability and expanding services to include adults and other medical conditions to benefit a larger population. Participants also proposed a reimbursement model for TCS provided within the community.

Our study addresses the gap in health care accessibility by establishing teleconsultation infrastructure within the school platform. Digital health services represent a clear solution to enhance health care access in remote areas [8-11].

This study exemplifies offering a solution through TCS to a remote semi-urban community in Karachi. It assesses the community’s health needs and evaluates the feasibility and experience of TCS in this context. Similar to other transitioning regions, Pakistan encounters challenges in ensuring equitable and accessible health care distribution to its expanding population, particularly those residing in villages and remote areas [3,4]. Our study demonstrated that TCS effectively meets the health care needs of the underprivileged in resource-challenged countries such as Pakistan. The quantitative arm of this study revealed that children of varying ages and with diverse health issues benefited significantly from this service. However, the qualitative arm of the study informed that the same services should be expanded to the adult population with various health specialties. Furthermore, having TCS available within school premises prevented children from missing school days to visit doctors located far from their homes for health issues. This increased their attendance and improved their overall attitude toward education and learning.

Furthermore, TCS minimized the costs associated with high consultation fees and transportation to visit a doctor in person. Caregivers were able to remain on campus, reducing lost wages. Literature also supports the role of school-based digital health solutions in reducing school absenteeism and saving costs related to external health care visits [15]. Furthermore, the literature suggests significant time and cost savings for patients receiving care for chronic and infectious diseases through digital health solutions in remote regions of low- and middle-income countries (LMICs) [16]. This study not only documented the use of TCS for initial visits but also highlighted its use for follow-up visits until students fully recovered. During the data collection period, the majority of cases were successfully managed using TCS, with only a few requiring referrals to hospital services. This approach minimized the costs traditionally incurred by caregivers for travel to health care facilities in urban areas. Furthermore, timely referral is critical in predicting the outcome of severe illnesses.

### Comparison With Previous Work

One of the crucial aspects of this project is the integration of TCS within the school system. Numerous studies in the literature strongly support this integration [17]. Community stakeholders appreciated the integration of TCS within the school system for several reasons, including timely consultations, access to expert opinions, effective illness management, improvement in child health status, and cost-saving benefits.

Regarding the TCS fee model, our proposed solution remained significantly more affordable compared to visiting a local general practitioner. A similar example of low-cost TCS can be found in the literature, where the weekly costs for eye-related issues during COVID-19 were 56 times lower. This illustrates the cost-saving aspect of TCS while ensuring the safety of all individuals involved, thanks to its digital health care services [18]. Further exploration of the fee structure model for teleconsultation is necessary to ensure it is appropriately contextualized for different settings and populations.

This prototype model for the teleconsultation initiative has opened avenues to explore the scope of proactive digital health solutions in resource-constrained settings, including preparation for future epidemics. The impact of this project is 2-fold; first, this TCS school health model has now been owned and taken over by the provincial government for its sustainability. Second, the role of the school health nurse has been enhanced as a student counselor to cater to students’ psychosocial issues.

### Implications and Suggestions for Further Studies

Health care access remains a significant but often overlooked issue, and it is crucial for policy makers to acknowledge the pivotal role of health care workers and community health nurses as frontline personnel in achieving health-related goals in underprivileged areas [1-3]. Digital health modalities for delivering online health services have garnered significant attention from primary care practices during this unprecedented pandemic [8]. The US Center for Medicare and Medicaid Services approved teleconsultation services in 1997. However, widespread implementation by primary health care systems occurred during recent pandemics and major natural disasters worldwide, when physical access to health care became challenging [19].

Telehealth through TCS is a vast platform with immense scope for innovation. It uses technology to deliver health services, including awareness sessions, assessments, monitoring, and educational sessions for home health care, all under the guidance of medical experts. This approach addresses significant concerns related to health care access in underserved populations [14,16].

The implementation of TCS will reduce travel costs for patients and health care seekers. Furthermore, it will alleviate the burden on the health care system by ensuring equitable allocation of resources. TCS will also decrease unnecessary hospital visits, allowing health care costs to be redirected toward the delivery of high-quality care [20]. TCS can implement a multidisciplinary team model involving nurse practitioners, physicians, psychologists, and other health care workers, provided that human resources management is effectively handled. TCS enhances the role of the school health nurse as a crucial member of this interdisciplinary team. The nurse’s role includes independent practice in identifying issues, triaging and referring patients, and effectively applying clinical judgment, skills, and evidence-based nursing practice to assess, plan, implement, and evaluate treatment outcomes.

Literature supports the pivotal role of registered nurses in the community who contribute significantly to building team-based partnerships aimed at addressing health issues within their community [21].

In this digital era of technology, including smartphones, nurse-led TCS should be encouraged to support population health. Nurses can play a critical role in coordinating care, facilitating communication, promoting disease self-management, performing appropriate triage, and engaging in health promotion for families and communities. The accessibility and widespread use of smartphones further enhance the reach and effectiveness of nurse-led TCS, making health care more accessible and responsive to the needs of diverse populations [22]. Evidence globally has demonstrated promising outcomes of TCS in effectively managing diseases through online consultations. These consultations use audio, visual, graphics, and other electronic formats to deliver health care remotely, showcasing positive results in disease management across various regions [23]. Pakistan stands to benefit significantly from implementing TCS, which can help address challenges posed by unpredictable health and weather changes in the health care system. Policymakers and legislators can play an active role in allocating resources and developing policies to promote telehealth across various health care settings, including schools. This proactive approach can enhance health care accessibility, efficiency, and resilience in the face of evolving health and environmental conditions in Pakistan.

### Strengths and Limitations

This study’s greatest strength lies in the trust that TCS garnered from the community and stakeholders, leading to the establishment of a pioneering prototype model school offering TCS as a health care service in Pakistan. This model now has the potential to be replicated and scaled up in other community schools, with contextual adaptations based on the study’s findings. A cost-effective TCS infrastructure integrated into the school health room, along with a practical TCS model and networking with community stakeholders and school health experts, will significantly enhance health care access for the community and serve as a valuable asset for both the school and its surrounding community.

The use of a mixed methods study design not only helped identify consultation patterns but also captured caregiver preferences and experiences regarding this service, providing comprehensive insights.

However, the study’s limitation lies in its narrow generalizability, as it was conducted in only 1 government school in a semiurban setting, catering to children from ECD to Grade 8. This limits the broader applicability of the findings. In addition, the study’s small scale makes it challenging to assess the teleconsultation service’s overall impact on population health and community behavior change. Technical issues such as internet connectivity, technology maintenance, and sustaining a high-tech school health room also pose potential limitations for the ongoing viability of this model at the school.

### Conclusion

This study addresses the critical gap in health care accessibility by implementing TCS within the school platform. This initiative significantly influenced the perspectives of students and parents regarding the overall health of school children. Our findings underscored the effectiveness of integrating TCS, emphasizing its cost-effectiveness, time efficiency, and quality of service delivery. TCS integrated into schools reduced school absenteeism due to illness and alleviated financial burdens on families, aligning with global literature supporting digital health interventions.

Community feedback highlighted strong support for TCS, advocating for 24/7 availability, expansion to adult populations, and the implementation of a reimbursement model. Active involvement from policymakers and legislative support for resource allocation and policy development are crucial for the successful integration and sustainability of TCS. Policymakers should recognize TCS’s pivotal role in bridging health care gaps, particularly in underserved areas.

Led by school health nurses, TCS enhances health outcomes through improved coordination, disease management, and health promotion. With adequate resources and training, the prototype for TCS in school settings offers an innovative, scalable solution to health care access challenges, with significant potential to enhance health outcomes for school-going children not only in Pakistan but also in other resource-challenged settings, including LMICs.

## Abbreviations

ECD: early childhood development
ENT: ear, nose, and throat
FGD: focus group discussion
ICT: information and communication technology
LHV: Lady Health Visitor
LMIC: low- and middle-income country
MDG: Millennium Development Goal
NGO: nongovernmental organization
SDG: Sustainable Development Goal
TCS: teleconsultation services
